# Draft Genome Sequence of Fusarium equiseti K3, a Fungal Species Isolated from Hexachlorocyclohexane-Contaminated Soil

**DOI:** 10.1128/MRA.00885-21

**Published:** 2021-11-24

**Authors:** Nelson Khan, Rodolfo Brizola Toscan, Accadius Lunayo, Benson Wamalwa, Edward Muge, Francis J. Mulaa, René Kallies, Hauke Harms, Lukas Y. Wick, Ulisses Nunes da Rocha

**Affiliations:** a Department of Biochemistry, University of Nairobi, Nairobi, Kenya; b Department of Chemistry, University of Nairobi, Nairobi, Kenya; c Center for Biotechnology and Bioinformatics, University of Nairobi, Nairobi, Kenya; d Department of Environmental Microbiology, Helmholtz Centre for Environmental Research—UFZ, Leipzig, Germany; Vanderbilt University

## Abstract

We present the draft genome sequence of Fusarium equiseti strain K3, a fungus isolated from a hexachlorocyclohexane (HCH)-contaminated soil (Kitengela, Kenya). The 37.88-Mb draft genome sequence consists of 206 contigs, 12,311 predicted protein-coding sequences, and 261 tRNA sequences. This genome sequence contributes to our understanding of fungal-bacterial interactions during hexachlorocyclohexane degradation.

## ANNOUNCEMENT

The organochlorine pesticide hexachlorocyclohexane (HCH) was used for many years to control agricultural pests (1, 2). Despite a complete ban or severe restrictions on the use of HCH in many countries (3, 4), it continues to pose considerable environmental risks due to its toxicity, environmental persistence, and bioaccumulation in the food chain (2). HCH biodegradation as an effective bioremediation approach (5) has been studied extensively in bacteria (6) and white-rot fungi (7–9). At the same time, fewer data exist on degradation by non-white-rot fungi such as Fusarium species (2, 10).

**FIG 1:**
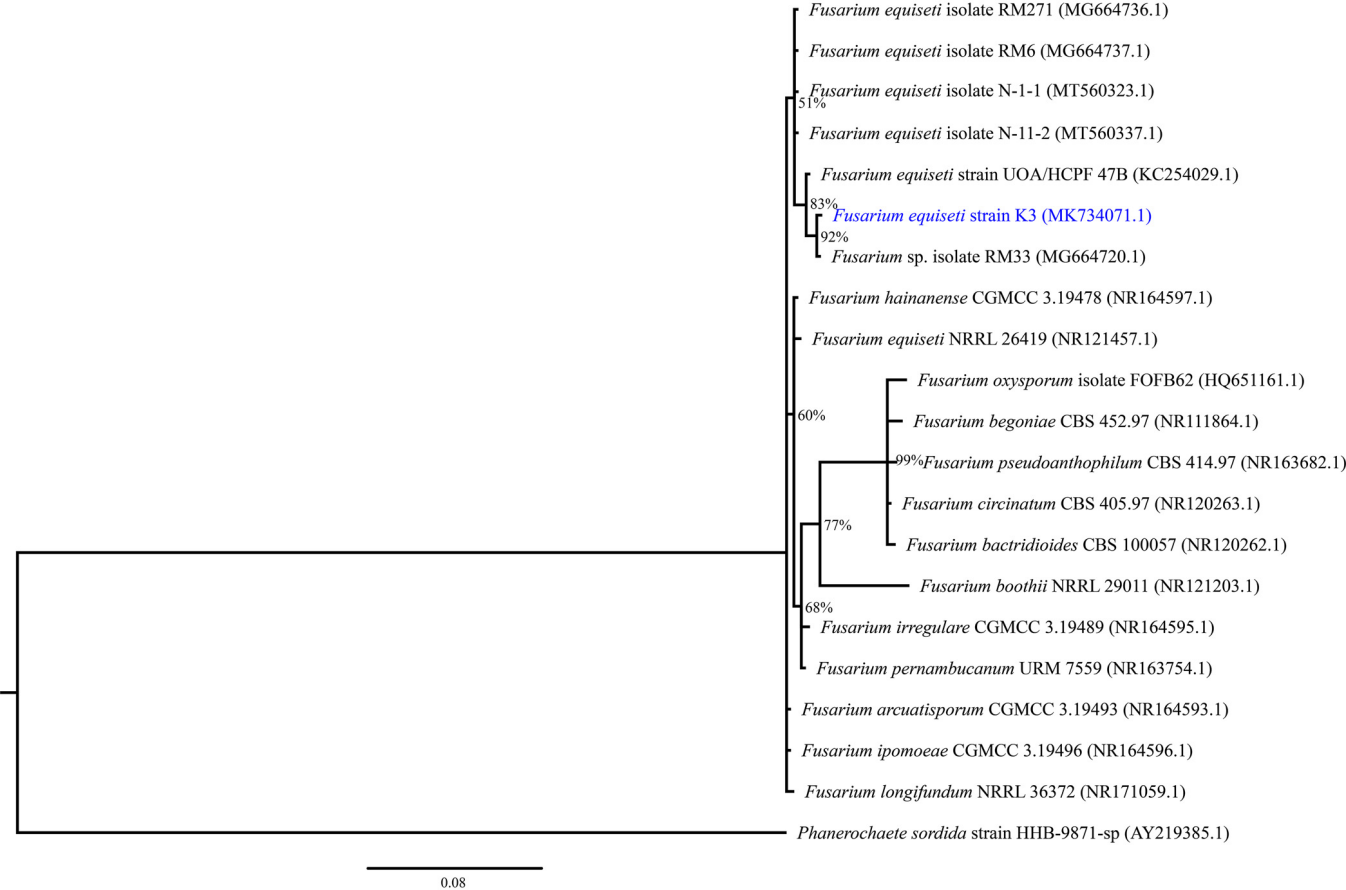
Phylogenetic tree showing the evolutionary relationship of Fusarium equiseti strain K3 (GenBank accession number MK734071.1) within the Fusarium *equiseti* clade, based on internal transcribed spacer 1 (ITS1), 5.8S rRNA gene sequences. The tree was constructed using MrBayes, a program for the Bayesian inference of phylogeny that is based on the Markov chain Monte Carlo (MCMC) method. Numbers at the nodes show percentages of posterior probabilities (derived from 1,000 samples), indicating the topological robustness of the tree. Phanerochaete sordida strain HHB-9871-sp was used as an outgroup to root the tree.

Here, we present the genome sequence of Fusarium equiseti strain K3, isolated from HCH-contaminated Kenyan soil from a former storage site at Kitengela, Kenya (01.49 S, 37.048 E), highly contaminated by organochloride pesticide (11). We identified our strain as *F. equiseti* based on a phylogenetic tree constructed using internal transcribed spacer 1, the 5.8S rRNA gene, and internal transcribed spacer 2 (complete sequence) and the large subunit rRNA gene (partial sequence) (Fig. 1). The fungus was isolated on minimum salt medium (MSM) agar plates (12) supplemented with 100 μg/ml *γ*-HCH in an inverted agar plate microcosm system described by Bravo et al. (13). An axenic fungal colony was obtained by subsequent repeated plating on 1:10 diluted potato dextrose agar supplemented with 100 μg/ml γ-HCH. The fungus’ low HCH degradation capacity was demonstrated in MSM medium as previously described by Sagar et al. (10).

Mycelium obtained from an agar plate overgrown with the fungus was used for DNA extraction using a Wizard genomic DNA purification kit (Promega, USA) and quantified applying a Qubit fluorometer (Thermo Fisher Scientific, USA). A NEBNext Ultra II FS DNA library prep kit (New England Biolabs, USA) was used to prepare a paired-end 300-bp library for genome sequencing on the Illumina MiSeq platform according to the manufacturer’s instructions, generating a total of 2,602,796 paired-end reads. We used Sickle v1.33 (14), with a Phred quality score of >30, to control quality and trim the sequences. *De novo* sequence assembly was performed using SPAdes v3.15.2 (15), while QUAST v5.0.2 (16) and BUSCO v5.0.0 (17), with the fungi_odb10 database, were used for a quality check and to provide completeness of the gene content within the assembly. Genome annotation was performed via the MAKER v2.31.11 pipeline (18) using AUGUSTUS v.3.4.0 (19), and SNAP v2013_11_29 (20) was used for *ab initio* gene prediction, with Fusarium graminearum PH-1 (GenBank accession number AACM00000000.2) as the training species. Unless otherwise stated, default parameter settings were applied for all software used.

The genome assembly of *F. equiseti* strain K3 resulted in 206 contigs with a total length of 37,882,472 bp (*N*_50_ contig length, 601,073 bp; GC content, 48.03%). The draft genome consists of 12,311 predicted protein-coding sequences. Analysis of the predicted protein-coding sequences using BUSCO (17) and the fungi_odb10 database (total of 758 genes) resulted in 749 (98.8%) complete single-copy genes, 3 (0.4%) complete duplicated genes, 3 (0.4%) fragmented genes, and 3 (0.4%) missing genes. A total of 261 tRNA genes were predicted using ARAGORN v1.2.36 (21). To gain insight into the *F. equiseti* strain K3 secondary metabolism, we used antiSMASH v6.0.0 (22) to predict the secondary metabolite biosynthetic gene clusters with the “fungi taxon” option. A total of 34 putative biosynthetic gene clusters previously reported for other Fusarium species were predicted. These included clusters likely to produce polyketides (23–25), terpenes (25, 26), and nonribosomal peptides (27–29).

The availability of the genome sequence of *F. equiseti* K3, together with ongoing efforts to understand its interactions with HCH-degrading bacteria, may provide invaluable insights into the use of fungal-bacterial cocultures for enhanced bioremediation of organic pollutants such as HCH (30).

### Data availability.

We deposited the *F. equiseti* K3 internal transcribed spacer 1, 5.8S rRNA gene, and internal transcribed spacer 2 (complete sequence) and large subunit rRNA gene (partial sequence) at ENA/DDBJ/GenBank under the accession number MK734071.1. We deposited the *F. equiseti* K3 whole-genome shotgun project at ENA/DDBJ/GenBank under the accession number CAJSTJ000000000.1. The version described here is the first version. The raw data are available at the ENA Sequence Read Archive (SRA) under the BioProject accession number PRJEB39686, BioSample accession number SAMEA7112172, and SRA accession number ERR4398881.
